# Alfaxalone anaesthesia increases brain derived neurotrophic factor levels and preserves postoperative cognition by activating pregnane-X receptors: an in vitro study and a double blind randomised controlled trial

**DOI:** 10.1186/s12871-022-01940-x

**Published:** 2022-12-24

**Authors:** Juliet M. Serrao, Colin S. Goodchild

**Affiliations:** Drawbridge Pharmaceuticals P/L, 23 Milton Parade, Malvern, Victoria 3144 Australia

**Keywords:** Alfaxalone, Allopregnanolone, Brain derived neurotrophic factor, Neuroprotection, Postoperative cognition, Pregnane X receptor

## Abstract

**Background:**

Alfaxalone is a fast acting intravenous anaesthetic with high therapeutic index. It is an analogue of the naturally-occurring neurosteroid allopregnanolone responsible for maintenance of cognition and neuroprotection by activation of brain pregnane X receptors and consequent increased production of mature brain-derived neurotrophic factor (m-BDNF). Two studies are reported here: an in vitro study investigated whether alfaxalone activates human pregnane X receptors (h-PXR) as effectively as allopregnanolone; and a clinical study that measured postoperative changes in serum m-BDNF and cognition in patients after alfaxalone anaesthesia compared with propofol and sevoflurane.

**Methods:**

In vitro Activation of h-PXR by allopregnanolone and alfaxalone solutions (206 - 50,000 nM) was measured using human embryonic kidney cells expressing h-PXR hybridised and linked to the firefly luciferase gene. Light emission by luciferase stimulated by each ligand binding with h-PXR was measured.

*Clinical* A double blind prospective randomised study of patients undergoing hip arthroplasty anaesthetised with alfaxalone TIVA (*n* = 8) or propofol TIVA (*n* = 3) or propofol plus sevoflurane inhalational anaesthesia (*n* = 4). The doses of anaesthetics were titrated to the same depth of anaesthesia (BIS 40-60). Subjects’ cognitive performance was assessed using the Grooved Pegboard Test, Digit Symbol Substitution Test (DSST) and Mini Mental State examination (MMSE) for 7 days postoperatively. Serum m-BDNF concentrations were measured for 7 postoperative days.

**Results:**

In vitro Allopregnanolone and alfaxalone both activated h-PXR, alfaxalone being more efficacious than allopregnanolone: 50,000 nM, *p* = 0.0019; 16,700 nM, *p* = 0.0472; 5600 nM, *p* = 0.0031.

*Clinical* Alfaxalone treated subjects scored better than propofol and sevoflurane anaesthetised patients in the cognition tests: (MMSE *p* = 0.0251; Grooved Pegboard test dominant hand pre v post anaesthesia scores *p* = 0.8438 for alfaxalone and *p* = 0.0156 for propofol and propofol/sevoflurane combined). The higher cognition scores were accompanied by higher serum m-BDNF levels in the alfaxalone anaesthetised patients (*p* < 0.0001).

**Conclusions:**

These results suggest that sedation and anaesthesia induced by the synthetic neuroactive steroid alfaxalone may be accompanied by effects normally caused by physiological actions of allopregnanolone at PXR, namely, increased secretion of m-BDNF and consequent neuroprotection and preservation of cognition.

**Trial registration:**

The clinical trial was registered on 17/01/2018 with the Australian New Zealand Clinical Trials Registry: registration number ACTRN12618000064202 [Universal Trial Number U1111-1198-0412].

## Background

Allopregnanolone is a metabolite of progesterone synthesized in the central nervous system where it promotes neurogenesis, neuroplasticity, and neuroprotection [2, 3]. Allopregnanolone has sedating and anaesthetic properties by virtue of being a positive modulator at gamma aminobutyric acid receptors type A (GABA_A_). The neuroprotective effects of allopregnanolone are due to activation of pregnane X receptors (PXR; NR112) and they are independent of its GABA_A_ receptor actions [4].

PXR is a nuclear receptor that binds with, and is activated by, a variety of xenobiotic and endogenous compounds, including naturally occurring steroids in the central nervous system, pregnenolone, progesterone and allopregnanolone [5, 6]. Consequent activation of PXR in the brain stimulates the production of mature brain derived neurotrophic factor (m-BDNF) a trophic factor accepted for its role in neurogenesis, synaptogenesis, and neuroprotection [7–9].

**Fig. 1:**
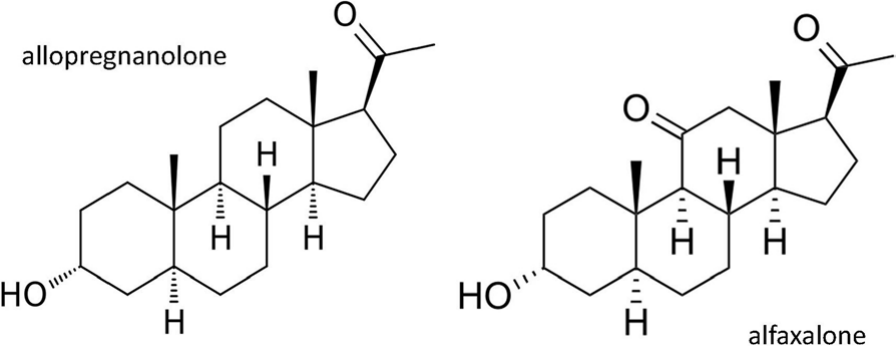
Allopregnanolone and alfaxalone: molecular structures compared. Alfaxalone is an analogue of the endogenous neurosteroid allopregnanolone

Alfaxalone, (3α-hydroxy-5α-pregnane-11,20-dione) is a synthetic pregnane steroid with potent anaesthetic and sedative properties by actions at GABA_A_ receptors [10]. It is an allopregnanolone analogue (Fig. 1) devoid of progestogen or endocrine hormonal activity [11].

An aqueous formulation of alfaxalone (Phaxan®; Drawbridge Pharmaceuticals, Malvern, Victoria Australia) has been developed for use as an intravenous sedative and anaesthetic [12]. It is hitherto unknown whether alfaxalone activates human pregnane X receptors (h-PXR), although its structural similarity to allopregnanolone suggests that it may do so. If alfaxalone activates the mechanisms utilized by allopregnanolone at h-PXR, including the increased production of m-BDNF, there would be implications for the use of neurosteroids such as alfaxalone in clinical anaesthetic and intensive care practice with respect to neuroprotection and preservation of cognition. Two studies are reported here with the combined aim of defining whether alfaxalone activates h-PXR and further whether alfaxalone anaesthesia is accompanied by effects predicted by h-PXR activation, namely preservation of m-BDNF secretion and postoperative cognition. The studies are:

- an in vitro study to compare equimolar concentrations of allopregnanolone and alfaxalone for efficacy in activating h-PXR using an in vitro assay of agonist binding to h-PXR and
- a clinical study in patients having hip joint replacements, in which the changes in serum m-BDNF levels and postoperative cognition were measured for 7 days in one group of patients after alfaxalone anaesthesia and in a comparator group that received propofol and sevoflurane as anaesthetic agents. This was an open-ended pilot study to assess feasibility of a new protocol evaluating a new intravenous anaesthetic, incorporating an assessment of postoperative cognition and associated serum m-BDNF.

## Methods

### In vitro assay platform

The activation of h-PXR by allopregnanolone and alfaxalone was measured by Indigo Biosciences, Inc., using Human Pregnane X Receptor (h-PXR) Assay Kit (supplier: Indigo Biosciences, Inc., 3006 Research Drive, Suite A1, State College, PA 16801, USA; + 1 (814) 234-1919). This consisted of suspensions of “reporter cells” in multi well plates. These were human embryonic kidney cells (HEK293) engineered by Indigo to express hybridised h-PXR in which the N-terminal sequence encoding the binding domain was substituted with yeast GAL4-binding domain functionally linked to firefly luciferase. The ligand binding and C-terminal domains of the h-PXR remained intact and functional. Thus, the effect of ligand binding (alfaxalone and allopregnanolone) with and consequent activation of h-PXR caused concentration dependent increase in luciferase activity and light emission from reporter cells exposed to the ligand. That luminescence by the reporter cells in each well plate was measured as relative light units (RLU) using a plate-reading luminometer.

### Test compounds for the in vitro study

The test compounds alfaxalone and allopregnanolone (Fig. 1) were obtained from Sigma-Aldrich (400 Summit Drive, Burlington, MA, USA 0180).

### In vitro assay methods

A suspension of reporter cells was prepared in cell recovery medium. Allopregnanolone and alfaxalone were diluted in dimethyl sulfoxide (DMSO) to generate concentrated stock solutions (1000 x the highest test concentration). These were diluted into screening medium containing 10% charcoal stripped foetal bovine serum to generate allopregnanolone and alfaxalone treatment solutions: 100,000; 33,333; 11,111; 3704, 1235; and 412 nM, respectively. One hundred microliter aliquots of treatment solutions were dispensed into quadruplicate assay wells, each well containing 100 μL suspension of reporter cells. Thus, each well contained the desired final treatment concentrations as shown in Table 1. Dimethyl sulfoxide concentration in all assay wells was 0.1%.

**Table 1:** Human Pregnane X Receptor Assay Results: The luminescence (expressed as relative light units) caused by a range of concentrations of allopregnanolone and alfaxalone; four wells at each concentration. Background luminescence caused by 0.1% solution of dimethyl sulfoxide used to dissolve the neurosteroids is also shown

Compound	Concentration (nM)	Luminescence (relative light units)			
**alfaxalone**	206	937	579	701	655
	617	869	733	873	617
	1852	937	971	915	707
	5556	1431	1461	1475	1293
	16,667	3123	2307	2747	2555
	50,000	4947	6074	5385	4637
**dimethyl sulfoxide**	0.10%	average of 6 wells = 666			
**allopregnanolone**	206	773	743	779	851
	617	739	763	789	725
	1852	715	811	775	675
	5556	1103	905	951	885
	16,667	2021	1983	1865	2101
	50,000	2117	2201	1875	2471

Assay plates were incubated for 22-24 hours (37 °C / 5% CO_2_ / 85% humidity). Following incubation, the treatment media were discarded and 100 μL of luciferase detection reagent (Indigo Biosciences) was added to each well. The luminescence (relative light units) was measured in each well using a luminometer. The luminescence recording from each well (RLU) was entered into an Excel spreadsheet. The readings were combined for each agonist (ligand) and concentration, and concentration/activity response curves were plotted (non-linear curve fitting software, GraphPad Prism version 9.4.1; GraphPad Software, 2365 Northside Dr. Suite 560, San Diego, CA 92108).

### Clinical study

The study protocol was written by the authors (JMS and CSG, Drawbridge Pharmaceutical P/L) and the clinical study was performed following cGCP guidelines, under contract to Drawbridge by staff at the Clinical research organisation at the Royal Adelaide hospital (PARC Clinical Research, Royal Adelaide Hospital, Port Road Adelaide SA 5000; principal investigator Prof Guy Ludbrook). The study protocol was approved by the Royal Adelaide Hospital human research ethics committee (approval number HREC/17/RAH/523) and also registered with the Australia and New Zealand Clinical Trials Registry. The study was designed as a pilot for larger planned Phase 3 studies to test the viability of the protocol and ability to differentiate between alfaxalone and established agents, propofol and sevoflurane used for anaesthesia during hip replacement surgery, with respect to two major outcomes: cardiovascular stability; and postoperative cognition with associated serum m-BDNF levels. Forty participants were randomized into 2 blocks of 20, each containing 10 subjects for group alfax_TIVA (alfaxalone induction and alfaxalone infusion maintenance) and 10 for group prop/sevo (5 subjects propofol induction and propofol infusion maintenance; 5 subjects propofol induction and sevoflurane maintenance). Treatment randomisation was performed in pharmacy and the list maintained there, to be released to PARC at the interim analysis planned after 20 subjects had completed the study and at the conclusion of the study. All drug dispensing was performed by the pharmacy clinical trials unit at the Royal Adelaide Hospital. The trial drugs were supplied to the operating theatre in a sealed box and the nature of the anaesthetics used in each case was concealed from staff making and recording peroperative and postoperative measurements. The anaesthetist administering the anaesthetics was aware of which anaesthetic was being used but the patient and the staff making postoperative measurements of cognition were unaware of which anaesthetic was administered. The trial was stopped at *n* = 15 because recruiting and surgery in the orthopaedic unit at the Royal Adelaide Hospital ceased for several months when there was an outbreak of MRSA infections. The data presented here are from those 15 subjects. The trial was not recommenced because the analysis of the data from the pilot study (reported here) answered the qustions on viability of the protocol and its ability to provide meaningful comparisons with respect to cardiovascular stability and postoperative cognition/m-BDNF levels.

Subjects were not admitted to the study if they scored badly on the National Adult Reading Test, or if the Hospital Anxiety and Depression scale indicated clinical depression or anxiety. Subjects were also excluded from the study if they had preoperative cognitive delay (a score less than 24 out of 30 in the Mini Mental State Examination (MMSE)).

The subjects enrolled in the study were studied from 16th April 2018 to 27th February 2019. Informed written consent was obtained from 15 patients prior to enrollment into this prospective double-blind randomized study of anaesthesia for hip replacement surgery. The test group (*n* = 8) received alfaxalone total intravenous anaesthesia (alfax_TIVA) and the comparator group (prop/sevo) received either propofol induction followed by sevoflurane inhalational anaesthesia (*n* = 4) or propofol total intravenous anaesthesia (*n* = 3). Fentanyl for analgesia and cis-atracurium for muscle relaxation were administered as indicated clinically. Anaesthetic drug dosing was titrated to maintain bispectral index (BIS) at 40-60. Intravenous fluid replacement and metaraminol were given to maintain a normal blood pressure within 20% of pre-anaesthetic values. Respiratory parameters (end-tidal carbon dioxide and arterial oxygen saturation), and core temperature were all maintained within normal physiological limits.

Subjects were tested for level of cognition with the Grooved Pegboard Test, Digit Symbol Substitution test (DSST), and MMSE preoperatively and again postoperatively on day 7 (pegboard test and DSST) or daily for up to 7 days (MMSE). Blood was withdrawn from each subject into a serum sampling tube for the measurement of m-BDNF prior to anaesthesia induction, 30 minutes after the start of surgery, at the end of surgery and then daily each morning for up to 7 days postoperatively. The blood samples were allowed to clot for lh at room temperature and lh at 2 °C to 8 °C followed by centrifugation at 2000 g for l0min at 4 °C. Serum samples were decanted and then stored at − 80 °C. Analysis for m-BDNF concentrations was performed by TetraQ, Royal Brisbane and Women’s Hospital, Level 7 Block 6, Herston, Queensland 4029, Australia using a commercial human brain-derived neurotrophic factor (BDNF) ELISA Kit (Aviscera Bioscience INC. 2348 Walsh Ave STE C, Santa Clara, CA 95051, United States; Cat# SK00752-01). The specificity of this test was 100% for human m-BDNF and < 1% for human pro-BDNF. The mean accuracy of each standard between batches range was − 1.0 to 2.6%, while the precision of the standards was between 1.8 and 8.8%.

All statistical comparisons were made with GraphPad Prism version 9.4.1 for Windows, GraphPad Software, San Diego, California USA, www.graphpad.com.

## Results

### In vitro study

**Fig. 2:**
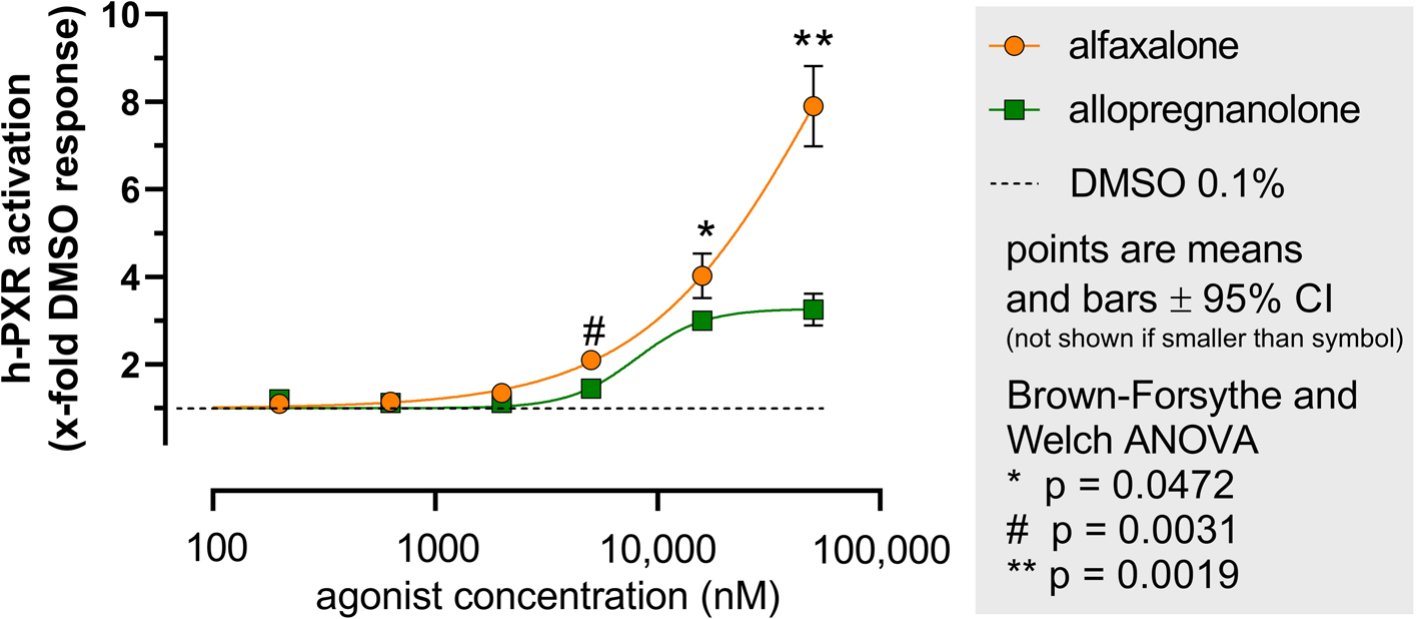
Ligand log concentration dose response curves: human pregnane X receptor activation. Luminescence caused by ligand interaction with h-PXR in each well was expressed as a multiple of the average luminescence caused by 0.1% dimethyl sulfoxide vehicle controls (*n* = 6). Alfaxalone is more efficacious than allopregnanolone in activating h-PXR at: 50,000 nM [**, *p* = 0.0019]; 16,700 nM [*, *p* = 0.0472]; and 5600 nM [#, *p* = 0.0031]. The maximum values at the top of the log concentration response curves, by 50,000 nM solutions were: 3.25, 2.66 – 3.83 (allopregnanolone mean, 95% CI); 7.90, 6.44 – 9.36 (alfaxalone mean, 95% CI)

Allopregnanolone and alfaxalone both produced clear colourless aqueous solutions up to maximum concentration of 50,000 nM in 0.1% dimethyl sulfoxide. Testing higher concentrations of drug was not possible because the higher concentrations of dimethyl sulfoxide necessary to achieve drug dissolution would have disrupted normal functions of the reporter cells. Both pregnane steroids activated the hybrid h-PXR to cause concentration-related light production by the linked firefly luciferase (Table 1 and Fig. 2).

Statistical comparison using Brown-Forsythe and Welch ANOVA (GraphPad Prism version 9.4.1) confirmed alfaxalone was more efficacious than allopregnanolone in activating h-PXR at three concentrations: 50,000 nM [*p* = 0.0019]; 16,700 nM [*p* = 0.0472]; and 5600 nM [*p* = 0.0031]. The values at the top of the log concentration response curves showing the activity caused by the 50,000 nM solutions of both ligands were: 3.25, 2.66 – 3.83 (allopregnanolone mean, 95% CI); 7.90, 6.44 – 9.36 (alfaxalone mean, 95% CI). The allopregnanolone curve reached a plateau at concentrations 16,700 nM and 50,000 nM but the alfaxalone curve did not. Testing higher concentrations of alfaxalone to reveal the plateau portion of the concentration response curve was not possible because of limitations in dissolving higher concentrations of alfaxalone in 0.1% DMSO.

### Clinical study

No subject scored poorly on the National Adult Reading Test prior to the study. All subjects scored 24 or higher on the MMSE prior to study entry, indicating normal cognitive ability, and none were clinically anxious or depressed as indicated by the Hospital Anxiety and Depression scale. The alfax_TIVA group and the comparator group (prop/sevo) were equivalent for age range, gender balance, score in ASA Physical Status Classification System and for duration of surgery and anesthesia (Table 2).

**Table 2:** Alfaxalone-treated subjects (alfax_TIVA; *n* = 8) compared with subjects treated with propofol and sevoflurane (prop/sevo; *n* = 7). The groups were comparable with respect to the parameters of age, gender mix, ASA classification, weight and duration of surgery and anaesthesia. When the blood pressure measurements taken during surgery and anaesthesia were compared statistically with BP systolic measurements taken before anaesthesia there was a significant decrease in prop/sevo subjects but not alfax_TIVA subjects. However, the mean BP systolic during anaesthesia was 148 mmHg for alfaxalone-anaesthetised subjects and 131 mmHg in propofol and sevoflurane-anaesthetised subjects; no clinically-significant hypotension occurred in either group who recorded preoperative values that were normal and similar (159, 15.9; 158, 13.9: mean, 95% CI mmHg.). All parameters derived from the BIS measurements indicate that the doses of the anaesthetics caused the same onset, control of depth at target level (BIS 40-60), smoothness of control and speed of recovery

parameter	alfax TIVA		prop/sevo				
age (yrs; mean, range)	68.3	48 - 78	66.1	54 - 74	**statistical testing exact** ***p*** **values are shown**		
M:F	5:3		5:2				
ASA 1:2:3	0:5:3		0:5:2				
wt (kg; mean, range)	88.1	73 - 119	99.7	70 - 126			
duration of surgery (hr; mean, range)	3.77	1.74 - 5.45	2.26	0.76 - 3.54			
duration of anaesthesia (hrs; mean, range)	4.40	2.31 - 6.27	2.86	1.25 - 4.34			
preop systolic BP (mm HG; mean, 95% CI)	159	15.9	158	13.9	0.2974	0.0007	paired t-test
intraop BP systolic (mm Hg; mean, 95% CI)	148	13.1	131	10.9			
time after induction to first BP support (mins; median, IQ)	54.00	35-157	7	4-34	0.0111		Mann-Whitney test
dose metaraminol during anaesthesia (mg/hr.; median, IQ)	1.87	0.71-2.26	2.13	1.19-2.90	0.379		
time to BIS< 60 after induction (minutes; mean, 95% CI)	1.44	0.39	1.36	0.41	0.79		unpaired t-test
recovery time to BIS 90: (minutes; mean, 95% CI)	18.69	12.46	14.50	11.71	0.64		
time to open eyes: (minutes; mean, 95% CI)	4.31	1.88	6.93	1.92	0.08		
BIS from 90 sec after induction to end surgery: BIS (mean, 95% CI)	43	3	48	4	0.06		
BIS coeff of variation from 90 sec after induction: (mean, 95% CI)	20	3	16	6	1.00		
% total anaesthetic time when BIS < 40 (mean, 95% CI)	28	17	16	15	0.33		
% total anaesthetic time when BIS > 60 (mean, 95% CI)	4	3	10	5	0.07		

The amount of iv fluid (crystalloid) replacement was 0.7 (0.14) and 0.78 (0.28) [mean (SD) l/hr] for alfax_TIVA and prop/sevo patients respectively. Table 2 also shows comparison of pre-anesthetic systolic blood pressure (BP) with the mean BP during anesthesia and surgery for both groups. Clinically significant hypotension did not occur in either group of subjects although there was a statistically significant reduction in systolic BP in the prop/sevo group (from a preoperative value of 158 mmHg. to mean systolic BP during surgery of 131 mmHg.) compared with preoperative 159 mmHg. to peroperative mean of 148 mmHg in alfaxalone-treated subjects. This was achieved with intravenous fluid replacement, which was the same in both treatment groups and the administration of the sympathomimetic drug, mataraminol, the doses of which were also similar in both alfax_TIVA and prop/sevo treatment groups. The doses of metaraminol administered for the whole period of surgery and anesthesia [mg/hour; median (IQ)] were 2.13 (0.71-2.75) for alfax_TIVA and 3.19 (2.81-4.62) for prop/sevo patients. Thus all hypotensive episodes were promptly treated so that BP was kept within normal parameters (± 20% of preoperative values) (Table 2).

In the period after anaesthesia induction but before surgery started (30 minutes), the BIS values (median, 95% CI) were 41, 44-47 for alfax_TIVA and 49, 49-52 for prop/sevo subjects respectively. The alfax_TIVA and prop/sevo subjects exhibited the same speed of onset and recovery from anesthesia, smooth anesthesia control with respect to BIS target level and coefficient of variability (Table 2).

**Fig. 3:**
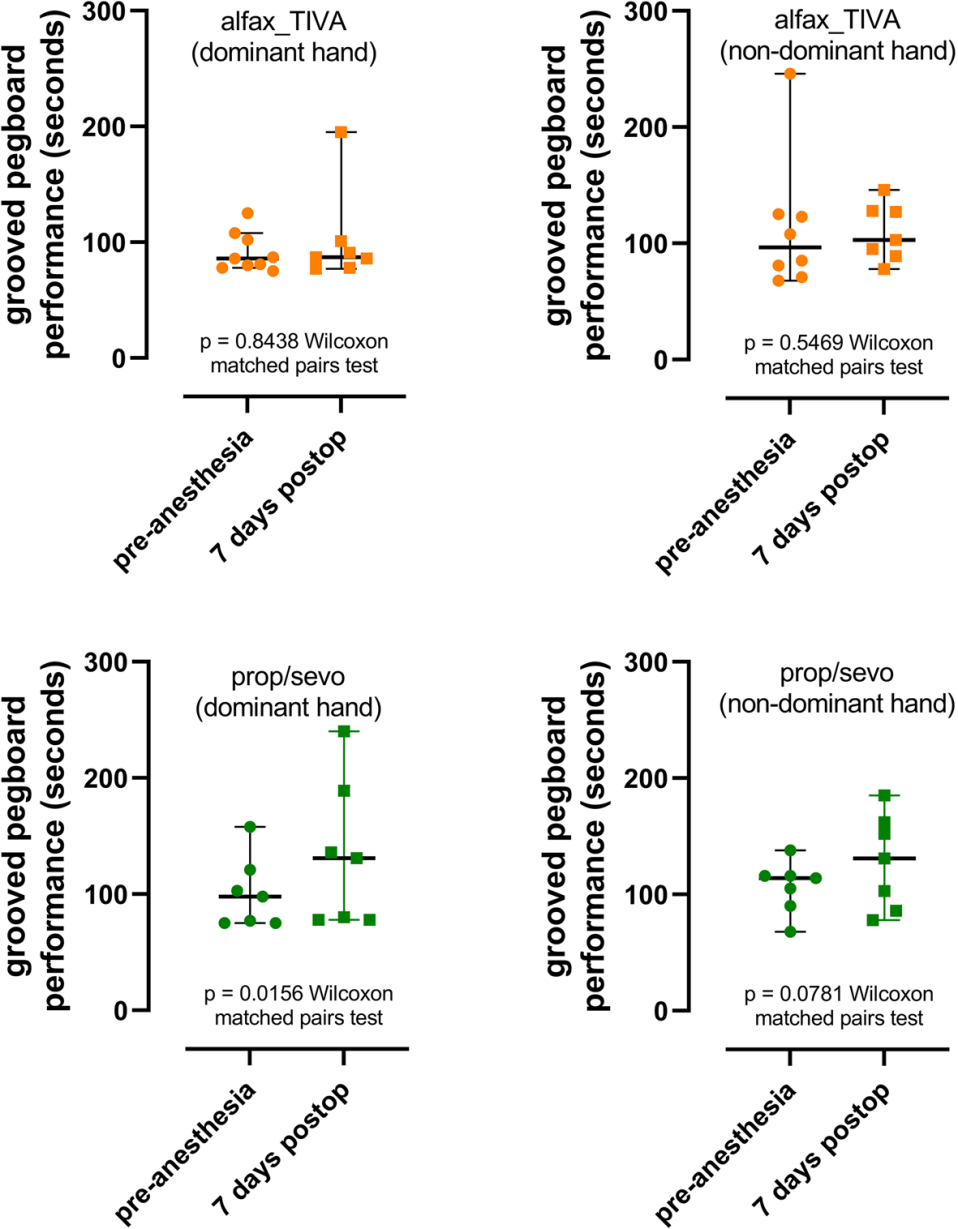
Grooved pegboard test measurements made preoperatively and 7 days postoperatively after alfaxalone and propofol/sevoflurane anaesthesia. Performance in this test did not decrease significantly after alfaxalone anaesthesia whereas performance was significantly poorer after propofol/sevoflurane anaesthesia. Points show individual measurements and bars, median and interquartile range

**Fig. 4:**
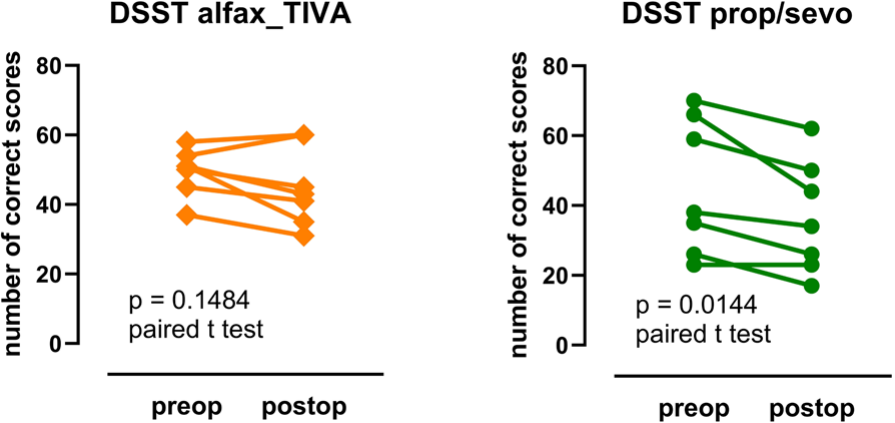
Digit Symbol Substitution Test (DSST) measurements made preoperatively and 7 days postoperatively after alfaxalone and propofol/sevoflurane anaesthesia (*n* = 7 subjects in each group). Performance in this test did not decrease significantly after alfaxalone anaesthesia whereas performance was significantly poorer after propofol/sevoflurane anaesthesia (*p* = 0.0144; paired t test). Points show individual measurements and lines connect the preop and postop measurements from the same individual subject

Alfax_TIVA-treated subjects scored better in the postoperative cognition tests. Grooved Pegboard Test and DSST performance scores preop compared with 7 days postop are shown in Figs. 3 and 4. Scores measured 7 days after surgery and anaesthesia compared with preoperative measurements were not different for alfaxalone anaesthetised subjects whereas the patients took longer to complete the pegboard task (*p* = 0.0156, dominant hand) and scored less correct answers in the DSST (*p* = 0.0144; paired t test) 7 days after surgery and propofol/sevoflurane anaesthesia.

**Fig. 5:**
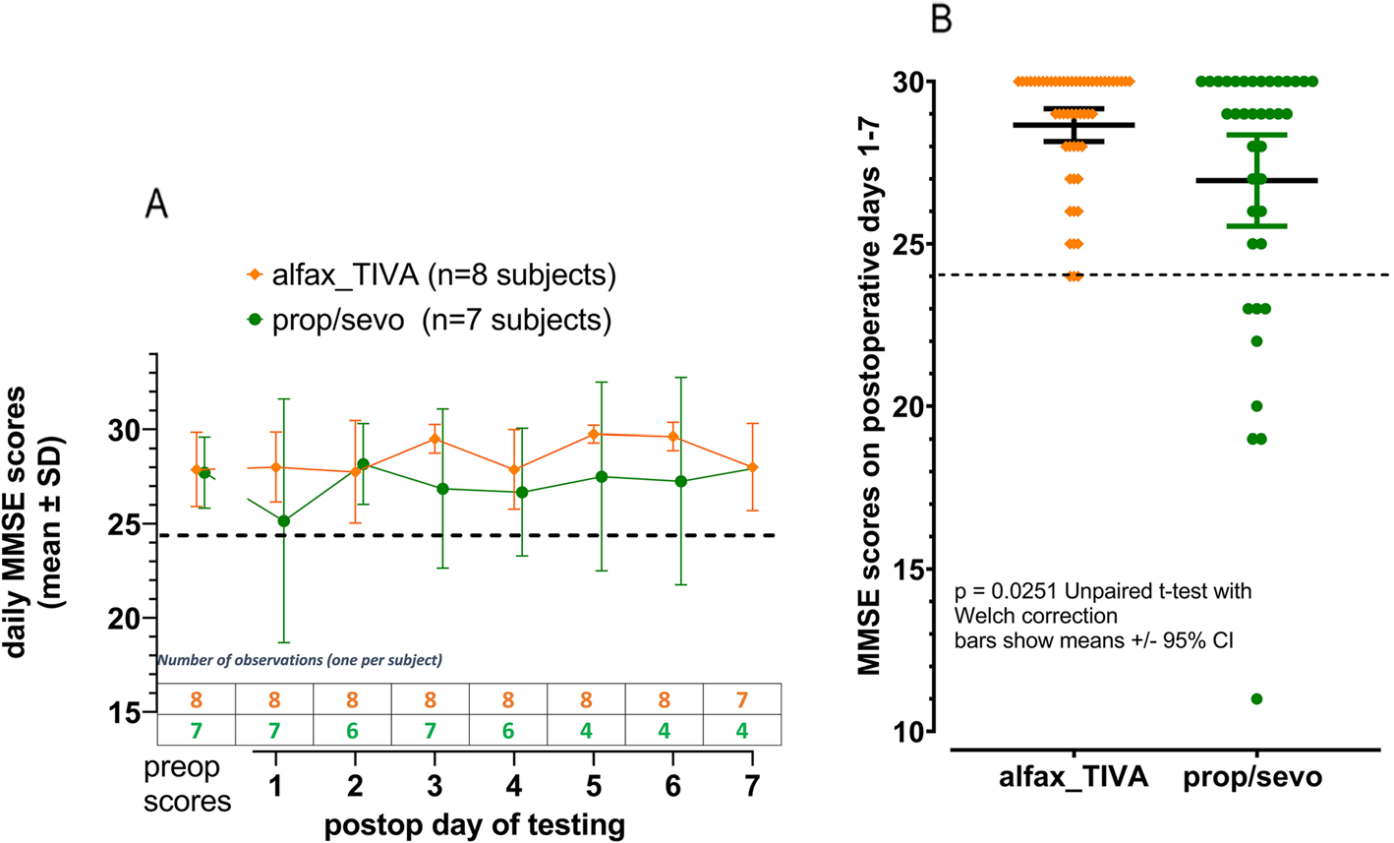
MMSE scores during 7 days postoperatively after alfaxalone anaesthesia (alfax_TIVA) and anaesthesia with propofol and sevoflurane (prop/sevo). Panel **A** shows means (± SD) for MMSE scores measured preoperatively and on days 1-7 postoperatively. Panel **B** shows all MMSE scores from all subjects measured on days 1 – 7 postoperatively. Only subjects anaesthetised with propofol and sevoflurane (prop/sevo) returned MMSE scores less than 24 indicated by the dashed horizontal lines

Alfax_TIVA-treated subjects also scored better in the MMSE compared with the prop/sevo group in the 7 days postop period (*p* = 0.0251; unpaired t-test with Welch correction, Fig. 5).

**Fig. 6:**
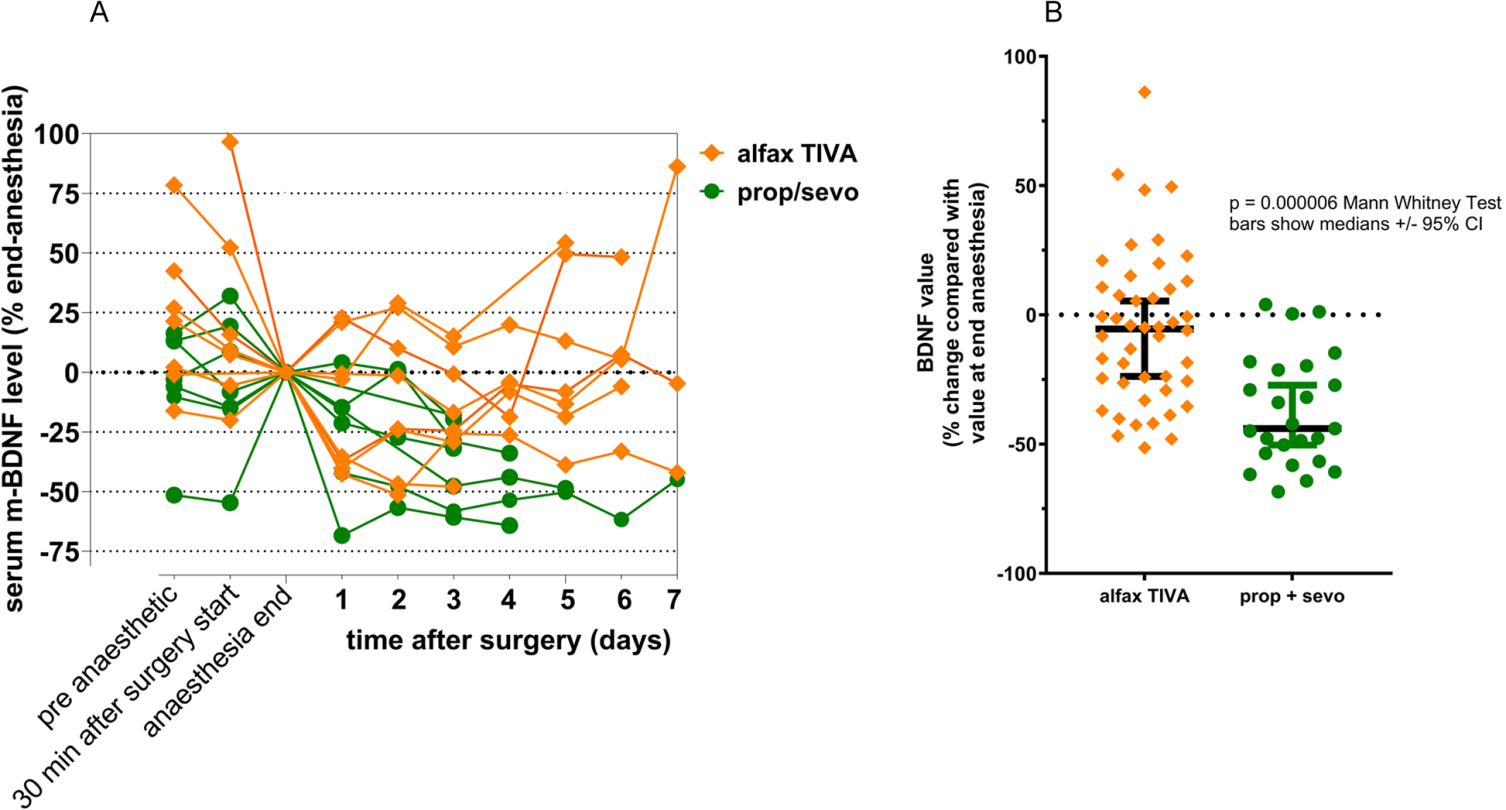
Serum m-BDNF levels measured in the 7 postoperative days after total hip joint replacement and anaesthesia with alfaxalone (alfax_TIVA; *n* = 8 subjects) or propofol and sevoflurane (prop/sevo; *n* = 7 subjects). Panel **A** shows BDNF values as a percentage reduction of the m-BDNF value obtained at the end of surgery and anaesthesia with alfaxalone (alfax_TIVA) and propofol and sevoflurane (prop/sevo) respectively. Points plotted in panel A are measurements from individual subjects and lines connecting the data points indicate measurements from an individual subject taken at different time points as indicated on the x axis. In panel **B**, the m-BDNF values are shown as % change compared with the levels at the end of surgery and anaesthesia. m-BDNF levels after prop/sevo anaesthesia were reduced by 44% (− 44, median; − 55 to − 20 (IQ)) compared with 5%, median reduction for alfaxalone treated patients (IQ range 27% reduction to 11% increase) [*p* = 0.000006; Mann Whitney Test]

The MMSE scores measured preoperatively were the same in both groups: 28 (30-25; 1.36), mean (range; 95%CI) in alfax_TIVA subjects; and 28 (29-25; 1.4), mean (range; 95%CI) in prop/sevo subjects (Fig. 5). All preoperative scores for individuals in both treatment groups were 24 or greater out of the maximum score of 30, indicating that no subject entering the study was cognitively impaired. Postoperative scores ≤23, indicating significant cognitive impairment after surgery and anaesthesia, occurred only in subjects anaesthetised with propofol and sevoflurane (prop/sevo) leading to a difference between the groups on statistical testing (*p* = 0.0251; unpaired t test with Welch’s correction (Fig. 5)). The better performance in the cognition tests in the first 7 days postop in alfax-TIVA subjects was accompanied by higher serum levels of m-BDNF in those patients compared with those anaesthetised with propofol and sevoflurane (*p* = 0.000006; Mann Whitney test, Fig. 6). The preoperative values for m-BDNF were 38,624 ± 8889 (mean, SD) pg/ml in the 7 prop/sevo subjects and 34,713 ± 9103 (mean, SD) pg/ml in the 8 alfax_TIVA subjects. The wide variability in m-BDNF levels necessitated standardisation of the data prior to statistical analysis. As shown in Table 2 above, the duration of surgery varied widely between individuals with a trend toward lengthier anaesthesia and surgery times in the alfax_TIVA group. Therefore, the m-BDNF levels measured at end surgery were used for standardisation of measurements made in the postoperative period. These are shown in Fig. 6.

## Discussion

There are many discussions around the topics of the possible beneficial (protective) and deleterious (neurotoxic) CNS effects of drugs used for sedation and anaesthesia in critical care practice. Although related, the two modalities of neurotoxicity and neuroprotection are different. The studies reported herein are concerned with possible neuroprotective effects of a compound being developed for use as a sedative and anaesthetic in critical care situations. Post synaptic GABA_A_ receptor interaction is the most common mode of action for anaesthetic and sedating drugs used in clinical anaesthesia and intensive care practice; for example, propofol, barbiturates, benzodiazepines, and inhaled drugs like isoflurane and sevoflurane. Unfortunately, those commonly used anaesthetic compounds have been shown to be neurotoxic, at least in immature brains [13–15], and the neurotoxic effects have been attributed to their interaction with GABA_A_ receptors. This seems to be at odds with the fact that neurosteroids, allopregnanolone and progesterone are neuroprotective compounds even though they are sedatives and anaesthetics that interact with GABA_A_ receptors [10]. Tesic et al. investigated this in preclinical models using alfaxalone and another neurosteroid analogue, (3α,5α)-3-hydroxy-13,24-cyclo-18,21-dinorchol-22-en-24-ol (CDNC24) [14]. They concluded that neuroactive steroid anaesthetics, including alfaxalone, were not neurotoxic in young brains, even though these compounds caused sedation and anaesthesia by interaction with GABA_A_ receptors [14]. They suggested those observations were not due simply to GABA receptor activity but the lack of neurotoxicity of the neuroactive steroids CDNC24 and alfaxalone may be at least partly related to suppression of presynaptic GABA release by these compounds in the developing brain [14].

The starting point for the studies reported herein, was that alfaxalone, a compound being developed for use as a sedative and anaesthetic in critical care, is not neurotoxic to young brains unlike the drugs used currently for those indications. The question was whether alfaxalone might also possess neuroprotective properties. Many critical care situations in which such a sedative/hypnotic might be used involve factors that cause damage to neurones and glia with functional consequences like those described as being caused by anaesthetic neurotoxicity: deficiencies in learning, memory, and cognition. Those factors are hypoxia, hypotension, stress, pain (surgery), sepsis, neurotrauma and pre-existing conditions such as neurodegenerative disease [16–19]. Alfaxalone, is a synthetic pregnane steroid. It is an analogue of allopregnanolone which is a metabolite of progesterone synthesized in the central nervous system where it promotes neurogenesis, neuroplasticity, and neuroprotection [2, 3]. The neuroprotective effects of allopregnanolone are due to activation of PXR and they are independent of its GABA_A_ receptor actions [4]. This mechanism has wide ramifications in critical care of not only patients with young brains but also those with degenerative disease including brains of older persons.

Mellon and colleagues showed neuroprotection 10 weeks after a single maximally effective dose of allopregnanolone on postnatal day 7 in mice with a progressive degenerative neurological disease called Niemann-Pick C [20–23]. Mellon postulated it is unlikely that allopregnanolone given at postnatal day 7, remains in high enough concentration, to continue to elicit GABA_A_-mediated effects 10 weeks later because allopregnanolone has a short half-life in plasma and the brain. The neuroprotective effect of allopregnanolone was also much larger than the effect of a synthetic neuroactive steroid, ganaxolone, even though ganaxolone is a much more potent positive modulator at GABA_A_ receptors than allopregnanolone. This result suggested to the authors that the neuroprotective actions of these neurosteroids may be due to a mechanism involving receptor sites other than GABA_A._ Further experiments revealed that treatment with allopregnanolone and its enantiomer, ent-allopregnanolone caused identical neuroprotection benefits even though ent-allopregnanolone, when compared with allopregnanolone, is a very weak GABA_A_ receptor positive modulator with minimal sedating and anaesthetic properties. The neuroprotective efficacy of the two enantiomers of allopregnanolone correlated with the ability of these compounds to activate PXR [4, 23]. It may be concluded from the aforementioned studies that endogenous neurosteroids, pregnenolone, progesterone and allopregnanolone bind with GABA_A_ receptors but also activate PXR in the central nervous system [2, 3]. The results of these actions are sedation, anaesthesia and neuroprotection, the latter being due to PXR activation.

The current studies support further investigation and clinical use of alfaxalone as a sedative/anaesthetic in critical care because it can cause neuroprotection as well as being devoid of neurotoxic activity. The in vitro study reports results that show the synthetic neuroactive steroid alfaxalone also activates h-PXR in a concentration dependent manner, and further, that it is a more efficacious ligand than allopregnanolone in this model. The concentration of alfaxalone needed to cause surgical anaesthesia in humans has been reported to be 4.48 to 0.98 mg/L [24]. When one calculates the concentration of free, non-protein bound alfaxalone (50% binding) the range of alfaxalone molar concentration during anaesthesia is 6740 to 1460 nM; in the range of PXR-activating concentrations shown in Fig. 2. These in vitro concentrations for h-PXR activation are also in agreement with observations of the GABA_A_ receptor positive modulation of alfaxalone in vitro patch clamp studies [10]. Alfaxalone (30 nM to 1000 nM) reversibly and dose-dependently potentiated the amplitude of membrane currents elicited by locally applied GABA. This suggests that doses of alfaxalone that cause sedation and anaesthesia by actions at GABA_A_ receptors will also be accompanied by the physiological actions typically caused by allopregnanolone activation of PXR. PXR activation causes effects on the regulation and secretion of a signal trophic factor involved in neurogenesis and synaptogenesis; brain derived neurotrophic factor (BDNF) [8]. PXR activation also decreases microglial inflammation [16, 25]. These effects in turn protect neurones and glia from the deleterious effects of challenges such as stress, trauma, and hypoxia.

The second, clinical study reported herein showed in the first 7 days after hip joint replacement under general anaesthesia, that patients anaesthetised with alfaxalone TIVA had better postoperative cognition accompanied by preservation of m-BDNF production compared with propofol TIVA and propofol/sevoflurane-anaesthetised subjects. This occurred when all subjects in both treatment groups demonstrated no cognitive delay before surgery. All subjects came from the same preoperative population and the depth of anaesthesia (BIS) was the same in both test and comparator groups. Potential confounding variables were controlled and equal for both groups: exclusions from the study (abnormal NART, HAD, MMSE); same type and severity of surgery; control of physiological parameters between normal limits (arterial oxygen and carbon dioxide levels, arterial pressure, core body temperature); other drug treatment doses (same dose fentanyl analgesia, same dose muscle relaxant, atracurium, same volume and type of intravenous fluid management; equal average blood loss, and metaraminol for control of blood pressure. The remaining cause for better postoperative cognition accompanied by preservation of m-BDNF production in one group was the anaesthetic; alfaxalone treatment compared with propofol and sevoflurane.

BDNF exists in three isoforms in a state of dynamic balance: preproBDNF, proBDNF and m-BDNF. The balance of neurogenesis and neurone survival versus neural pruning and apoptosis depends upon which isoforms are predominant. The absolute and relative concentrations of these isoforms vary with age, developmental stage of the individual, and pathology. The precursor molecule, pre-pro-BDNF is divided by intracellular and extracellular cleavage into two proteins, pro-BDNF and m-BDNF, each of which has distinct targets and actions [26–28]. The m-BDNF binds with tyrosine receptor kinase B [TrkB] receptors, present at the surface membrane of neurones and glia. The m-BDNF/TrkB complex thus formed, homodimerizes and auto phosphorylates [29]. The phosphorylated m-BDNF/TrkB activates several enzymes such as Akt (also known as Protein Kinase B) which promote neurogenesis and neuronal survival [8]. By contrast pro-BDNF actions predominantly activate pathways using the pro-apoptotic p75^NTR^ receptor. Under conditions where m-BDNF/TrkB phosphorylation is diminished and pro-BDNF predominates, developmental neural pruning [30] or apoptosis follow [31]. It has been shown in a preclinical neurodegeneration model that allopregnanolone increases BDNF and the neuroprotective signals associated with m-BDNF secretion [9].

Glial mediated inflammation in the central nervous system is caused by many factors such as trauma, hypoxia, stress, and by β-amyloid accumulation in Alzheimer’s disease [16–18]. All of these are common co-morbidities that complicate surgery, anaesthesia, and intensive care. Persistent microglial inflammation has been shown to cause apoptosis by inhibiting the neuroprotective pathways activated by m-BDNF [19]. PXR activation by neurosteroids such as allopregnanolone inhibits microglial hyperinflammatory responses in the central nervous system via neuroimmune regulatory proteins, such as CD55 [16, 25].

The experiments reported herein show that alfaxalone is an efficacious ligand in activating h-PXR, and alfaxalone administration for anaesthesia and sedation is accompanied by better postoperative cognition, that may be caused by increased m-BDNF activity and inhibition of the microglial inflammation caused by surgical stress. This has implications for critical care where neuronal function and survival are under attack by trauma, stress, hypoxia, and pre-existing neurodegenerative disease. For example, anaesthetic drugs and surgery have been linked with acute delirium and subsequent long-term deficits in cognition in older persons; effects associated with low levels of BDNF [32, 33]. It is also important to note that several studies have shown that the drugs used for sedation and anaesthesia that have been reported to be neurotoxic, (perhaps by virtue of their activity at post-synaptic GABA_A_ receptors), also have been shown to interrupt the normal functioning of the PXR/m-BDNF system described above, causing neuronal dysfunction. This suggests an alternative explanation for neurotoxicity caused by commonly used anaesthetic drugs rather than their activation of GABA receptors. Propofol causes dose dependent neuronal cell death at clinically relevant concentrations through inhibition of m-BDNF induced Akt secretion [34]. This removes the normal BDNF-induced function of Akt which is suppression of glycogen synthase kinase-3 (GSK3) activity [34]. GSK3 causes mitochondrial fission and apoptosis, effects that increase if the normal Akt inhibition is reduced e.g., by propofol administration. This is also a mechanism cited for neurotoxicity of other compounds acting at GABA_A_ receptors i.e., sevoflurane, isoflurane, and at NMDA receptors, ketamine [35–37]. It is important to note that this explanation avoids the problem with the GABA receptor theory, that ketamine, which acts at NMDA receptors, causes similar neurotoxic effects. Propofol also increases apoptosis by its actions at the pro-apoptotic p75NTR [38]. In other studies, sevoflurane anaesthesia caused increased levels of pro-inflammatory cytokines in microglia, and a simultaneous decreased activation of the m-BDNF induced Akt pathway in both the cortex and hippocampus [39]. No such reports exist for alfaxalone, allopregnanolone or progesterone. Established thinking suggests neuroprotection and neurotoxicity of current anaesthetics is mediated at GABA_A_ receptors, their conventional site of action. This report would suggest that the neuronal well being produced by alfaxalone is due to action distanced from the site of its anaesthetic effects at GABA_A_ receptors.

However, the question remains as to whether the neuroprotection provided by neurosteroids is distinct and separate from their actions at GABA receptors and is exclusively due to its actions at PXR or the systems are related. Further investigation is warranted on this question. For example, recently it has been shown that low levels of BDNF have been accompanied by reduction in cell surface α5 subunit-containing GABA_A_ receptors in prefrontal pyramidal neurones with associated behavioural deficits that could be reversed by BDNF [40]. The data reveal a novel causal mechanism by which deficient BDNF leads to reduced GABAergic signalling through regulation of p62, an adaptor protein.

It has been suggested that anaesthetic induced neurotoxicity occurs at times of relative paucity of astrocytes and the m-BDNF they secrete [34]. These conditions occur in young and aged brains. Thus, adverse neurocognitive sequalae are more likely to occur when one or a combination of factors [surgery (trauma), toxins (anaesthetics), inflammation, superimposed injury] come together at vulnerable times of life when brain natural protective mechanisms are at their weakest. The implication of this notion is clear. Using alfaxalone for the anaesthetic or sedative during those times would tend to bolster the natural neuroprotective mechanisms by activating PXR and shifting the balance towards neurogenesis and neurone survival (secretion of m-BDNF) and away from neural pruning and apoptosis (secretion of pro-BDNF). By contrast, the use of anaesthetic drugs that compromise the PXR/BDNF neuroprotection processes as described above, would tip the balance towards adverse cognitive sequalae in situations in which natural protective mechanisms are at their weakest e.g., in the very young and elderly patient.

### Study limitations

The first study reported herein used in vitro methods, with the naturally occurring hormone, allopregnanolone being the active control for the test compound alfaxalone. This method is very different to a complex in vivo environment However, the naturally occurring compound, allopregnanolone was active in this model and alfaxalone was more efficacious than allopregnanolone in h-PXR activation under the same in vitro conditions. Allopregnanolone is known to be active in vivo in activating PXR and BDNF systems in the CNS [4]. The study reported here, although performed in vitro, suggests strongly that alfaxalone administration will be accompanied by the same effects of PXR/BDNF actions because alfaxalone was more efficacious than allopregnanolone in h-PXR activation; and it is well known that alfaxalone can cross the blood brain barrier to access these receptors in the brain because it has anaesthetic activity.

In vitro systems are, however, artificial. It is important to confirm in vitro findings using intact animal and human models. Data published by Yawno and colleagues showed that normal anaesthetic doses of alfaxalone can replace allopregnanolone in the control of apoptosis in foetal lambs [41]. This effect is explainable by the allopregnanolone/PXR/BDNF interactions referred to above.

There is a published study in cats that reported results consistent with the findings of the in vitro study reported herein. Cervantes and colleagues showed in a placebo-controlled study in cats, that a single anaesthetic dose of alfaxalone prevented severe neurological damage caused by 8 minutes cardiorespiratory arrest [42]. The alfaxalone was given intravenously after re-establishing normal circulation and respiration. The alfaxalone treated subjects had normal EEG and neurology in the week following this neurological challenge, whereas the untreated cats had persistent epileptic EEG patterns and significant neurological damage. The example concerns reperfusion injury in the central nervous system after hypoxic injury, in which microglial inflammation is well established as a pathological mechanism for neural damage [43]. Alfaxalone activation of PXR and consequent inhibition of microglial inflammation as described above is consistent with these observations.

The clinical study is limited by the small numbers of patients studied. This was due to the fact this was a pilot study to assess feasibility of a new protocol to assess a new intravenous anaesthetic, incorporating an assessment of postoperative cognition and associated serum m-BDNF. The interim analysis performed earlier than planned, after 15 instead of 20 completed subjects, proved to be robust in giving a clear result.

## Conclusions

The results of the in vitro studies reported herein show that alfaxalone binds with h-PXR. The published literature suggests that such an action can promote natural mechanisms that support m-BDNF function with the result of preserving neural functions and cognition [1, 41, 42]. The observations made in the clinical study are completely consistent with alfaxalone anaesthesia activating those mechanisms leading to higher levels of serum m-BDNF and improved cognition postoperatively. This observation may provide an answer to a clinical dilemma, namely, what drug should be used as anaesthetic or sedative in situations where the m-BDNF/TrkB system is under pressure to protect normal neural functions against the deleterious effects of stressors such as trauma, surgery, inflammation, hypoxia, and pre-existing neurodegenerative disease (Alzheimer’s)? That question is even more acute at the extremes of age when there is less capacity in the m-BDNF/TrkB system, the very young and the elderly. Currently used anaesthetics seem to compromise the natural m-BDNF mediated neuroprotection, whereas alfaxalone can support it.

## Data Availability

All data generated or analysed during the in vitro h-PXR study are included in this published article. The data sets for cognition and m-BDNF data shown in the clinical trial section of this paper are not available elsewhere.

## Abbreviations

BIS: Bispectral index
CNS: Central nervous system
DMSO: Dimethyl sulfoxide
EEG: Electroencephalogram
GABA_A_: Gamma aminobutyric acid receptors type A
GSK3: Glycogen synthase kinase-3
HEK293: Human embryonic kidney cells
h-PXR: Human pregnane X receptor
m-BDNF: Mature brain derived neurotrophic factor
NMDA: n-methyl-D aspartate
p75NTR: P75 neurotrophin receptor
PXR: Pregnane X receptors
Akt: Protein Kinase B
RLU: Relative light units
TIVA: Total intravenous anaesthesia
TrkB: Tyrosine receptor kinase B
